# IPF Fibroblasts Are Desensitized to Type I Collagen Matrix-Induced Cell Death by Suppressing Low Autophagy via Aberrant Akt/mTOR Kinases

**DOI:** 10.1371/journal.pone.0094616

**Published:** 2014-04-11

**Authors:** Richard Seonghun Nho, Polla Hergert

**Affiliations:** Department of Medicine, University of Minnesota, Minneapolis, Minnesota, United States of America; University of Bergen, Norway

## Abstract

Idiopathic pulmonary fibrosis (IPF) is a chronic, lethal interstitial lung disease in which the aberrant PTEN/Akt axis plays a major role in conferring a survival phenotype in response to the cell death inducing properties of type I collagen matrix. The underlying mechanism by which IPF fibroblasts become desensitized to polymerized collagen, thereby eluding collagen matrix-induced cell death has not been fully elucidated. We hypothesized that the pathologically altered PTEN/Akt axis suppresses autophagy via high mTOR kinase activity, which subsequently desensitizes IPF fibroblasts to collagen matrix induced cell death. We found that the autophagosome marker LC3-2 expression is suppressed, while mTOR activity remains high when IPF fibroblasts are cultured on collagen. However, LC3-2 expression increased in response to IPF fibroblast attachment to collagen in the presence of rapamycin. In addition, PTEN over-expression or Akt inhibition suppressed mTOR activity, thereby increasing LC3-2 expression in IPF fibroblasts. Furthermore, the treatment of IPF fibroblasts over-expressing PTEN or dominant negative Akt with autophagy inhibitors increased IPF fibroblast cell death. Enhanced p-mTOR expression along with low LC3-2 expression was also found in myofibroblasts within the fibroblastic foci from IPF patients. Our data show that the aberrant PTEN/Akt/mTOR axis desensitizes IPF fibroblasts from polymerized collagen driven stress by suppressing autophagic activity, which produces a viable IPF fibroblast phenotype on collagen. This suggests that the aberrantly regulated autophagic pathway may play an important role in maintaining a pathological IPF fibroblast phenotype in response to collagen rich environment.

## Introduction

Cell homeostasis is carefully regulated by two major systems in eukaryotic cells, the ubiquitin proteasome system and the lysosome. The lysosomal system is responsible for degrading macromolecules, including proteins, and for the turnover of cytoplasmic organelles by autophagy [1]–[2]. The autophagic pathway consists of several distinct steps, resulting in the sequestration of cellular cargo such as damaged organelles, protein aggregates, or pathogens by the double-membraned autophagosomes [3], [4]. The beneficial roles of autophagy are associated with the homeostatic turnover of damaged cellular organelles and proteins [5], [6]. However, it has been well established that deregulated autophagy is associated with several human diseases including cancer, neurodegenerative disorders, and inflammatory bowel diseases [6]–[10]. Prior studies have shown that stress inducing conditions such as ER stress, oxidative stress, or elevated ROS formation can promote the activation of autophagy [9], and that lung injury caused by hyperoxia, cigarette smoke, or toxins is thought to be the initial step toward autophagy in the pathogenesis of lung fibrosis [11], [12]. These studies further suggest that autophagy may be an important mechanism that represents an inducible response to stress in lung cells [13], [14]. Therefore, the current concept of the role of autophagy is that when cells are exposed to a stressful environment, autophagy is initially activated to protect the cells from these conditions until it reaches its threshold.

Idiopathic Pulmonary Fibrosis (IPF) is a progressive fibroproliferative lung disease of unknown cause. IPF is characterized by the accumulation of fibroblasts/myofibroblasts and aberrant remodeling of the lung architecture by excessive production of type I collagen rich matrix [15]–[18], [24]. Myofibroblasts are the main cell type that is responsible for this excessive production of extracellular matrix within fibroblastic foci that are characteristic of the fibrotic process. When normal lung fibroblasts attach to polymerized collagen, the PI3K/Akt pathway is suppressed by high PTEN activity, thereby inhibiting fibroblast proliferation and promoting apoptosis [19]–[21]. In contrast, when IPF fibroblasts attach to polymerized collagen, they exhibit high Akt activity due to PTEN suppression, thereby producing highly proliferative and anti-apoptotic phenotypes on collagen matrix [22]–[23]. Immunohistochemical analysis of IPF fibroblasts within the fibroblastic foci of IPF patient specimens also revealed that PTEN expression is suppressed while Akt activity is up-regulated [25]. Furthermore, prior studies have shown that autophagy is not activated in IPF fibroblasts, suggesting a possibility of a direct link between the deregulation of autophagy and the fibrotic process [41], [43]. We recently found that when IPF fibroblasts are cultured on polymerized collagen, autophagy is low, while normal lung fibroblast attachment to collagen increases autophagy induction. This finding suggests that normal lung fibroblasts sense polymerized collagen as a stress inducing environment while IPF fibroblasts have a pathologically altered mechanism to desensitize themselves to the stress inducing environment of polymerized collagen. Since Akt is an important kinase involved in negative regulation of autophagy via mTOR [26]–[28], [30], these data further suggest that the aberrant PTEN/Akt axis in IPF fibroblasts suppresses autophagy induction, thereby maintaining the pathological IPF fibroblast phenotype on polymerized collagen. Therefore, our goal is to verify whether aberrant autophagic activity desensitizes IPF fibroblasts to stress inducing environments and confers a cell death resistant IPF fibroblast phenotype on collagen rich matrix. We first examined autophagic activity by measuring the autophagosome marker, LC3-2 protein [29]. We found that when IPF fibroblasts were cultured on collagen matrix, LC3-2 expression was low. In contrast, LC3-2 expression in control fibroblasts was elevated when cultured on collagen matrix, suggesting that the induction of autophagy in IPF fibroblasts is suppressed under this condition. Furthermore, when PTEN was over-expressed or Akt/mTOR functions were inhibited, LC3-2 expression was increased in IPF fibroblasts. Moreover, when autophagic activity in IPF fibroblasts expressing either PTEN or dominant negative Akt was inhibited by autophagosome inhibitors 3-methyl adenine (3MA) and chloroquine (CQ), cell death was significantly increased. Finally, we found elevated p-mTOR expression while LC3-2 expression was low in cells within the fibroblastic foci of IPF patient lung specimens. These studies showed that IPF fibroblasts have acquired a desensitizing mechanism to respond to environmental stress such as polymerized collagen matrix by utilizing the PTEN/Akt/mTOR axis, and this pathologically altered autophagic pathway may be responsible for the IPF fibroblast's ability to maintain a viable phenotype on collagen rich matrix.

## Materials and Methods

### Ethics statement

This study involves the analysis of human IPF patient specimens. Primary fibroblast lines were obtained from unused, existing pathological human tissue samples, and therefore is exempt (exemption 4). Tissue samples were stripped of all identifiers and designated as waste. All patients underwent procedures for diagnostic or therapeutic procedures. Written informed consent was obtained on all patients prior to the procedure being performed. Use of human tissues was approved by the Institutional Review Board (IRB) at the University of Minnesota.

### Human subjects

Cell lines were derived from lungs removed at the time of transplantation or death. The diagnosis of IPF was supported by history, physical examination, pulmonary function tests, and typical high resolution chest computed tomography findings of IPF. In all cases, the diagnosis of IPF was confirmed by microscopic analysis of lung tissue and demonstrated the characteristic morphological findings of usual interstitial pneumonia. All patients fulfilled the criteria for the diagnosis of IPF as established by the American Thoracic Society (ATS) and the European Respiratory Society (ERS). For this study, new primary IPF fibroblast lines were generated as tissue became available. To reduce technical variability, we routinely utilize cells between passages 5 and 7 because of concern that the phenotype of the cells is altered at higher passage. To address concerns of biological variability, we studied 11 control cell lines and 10 IPF cells lines.

### Cell culture and type I collagen matrices

Primary control and IPF lung fibroblast lines were generated by explant culture and cultured in high glucose DMEM containing 10% FCS. Fibroblasts were used between passages five and seven. Cells were characterized as fibroblasts as previously described [22]. Three-dimensional polymerized collagen matrices (PureCol, Advanced BioMatrix, CA, final concentration  = 2 mg/ml) were prepared by neutralizing the collagen solution with a one-sixth volume of 6 × DMEM medium and diluting to a final volume with 1×DMEM, and incubating the solution at 37°C for 3 to 4 h before use.

### Cell viability and proliferation assay

To precisely measure viable cells on collagen matrix, Cell-Titer-Blue Cell Viability Assay (Promega, WI) was used. Briefly, 3×10^4^ of control or IPF fibroblasts expressing PTEN or Akt or empty vector were cultured on type I polymerized collagen coated 96 well plates in 100 µl of serum free medium for 24 h in the presence or absence of autophagy inhibitors as indicated. 20 µl of Cell Titer Blue reagent was then added to each well and further incubated for 1 to 2 h. Cell viability was measured by a 96 well plate reader with a fluorescence filter set (560(Ex)/590 (Em)). In order to verify whether low viability in control fibroblasts on collagen is due to toxic effects of 3MA and/or CQ, IPF and control fibroblasts were cultured on tissue cultured plates in the presence of various doses of 3MA and/or CQ, and cell viability was also measured. For the measurement of fibroblast proliferation, CellTiter 96 AQ_ueous_ One Solution Cell Proliferation Assay kit was used (Promega, WI). Briefly, 3×10^4^ IPF and control lung fibroblasts were grown on polymerized collagen in 100 µl serum free DMEM medium on 96 well plates overnight. 20 µl of CellTiter 96 AQueous One Solution reagent was then added to each well of the 96-well plate followed by incubation for 1 h at 37°C in a humidified 5% CO_2_ atmosphere. The absorbance at 490 nm using a 96 well plate reader was then measured.

### Antibodies, chemicals and adenovirus constructs

For Western analysis, PTEN (catalog No. 9559), Anti-Akt (catalog No. 9272) and phosphorylated ser 473 Akt (catalog No. 4060) antibodies were obtained from Cell Signaling Technologies, MA. Actin (catalog No Sc-130656) and GAPDH (catalog No. Sc-25778) antibodies were obtained from Santa Cruz Biotechnologies (Santa Cruz, CA.) LC3-2 antibody (catalog No. 3868) was purchased from Cell Signaling Technologies, MA. Phosphorylated ser 2448 mTOR antibody was purchased from Abcam (catalog No. 51044, MA.) For the autophagy inhibition assay, 3-methyladenine (3MA), and chloroquine (CQ) were purchased from Calbiochem (Rockland, MA) and InvivoGen (San Diego, CA) respectively. For PTEN adenovirus construction, wild type (WT) PTEN cDNA was generated from normal human lung fibroblasts by RT-PCR. The primers used for wild type PTEN, which span the entire coding region of PTEN are 5′-CTA CTC GAG GCT CCC AGA CAT GAC-3′ and 5′-ACG CTC GAG ATA AAA AAA AAT TCA G-3′. The lipid and protein phosphatase truncated mutant was generated by PCR with primers 5′ -CAG CTCGAGGGACGAACTGGTGTA A-3′ and 5′ -ACG CTC GAG ATA AAA AAA AAT TCAG-3′. The amplified products were cloned into MIGR1-IRESGFP vector, followed by SalI and EcoRI digestion. The wild type and mutant PTEN MIGR1-IRES-GFP fragments were then cloned into adenovirus vectors, pAxCAwt (purchased from Dakara Bio. Inc.). The recombinant adenovirus with wild type PTEN was generated and collected in HEK 293 cells according to the company's instructions. Adenovirus titer was measured by the plaque method in 0.5% soft agar. The cells were infected with adenoviral vectors at a multiplicity of infection of 1∶20. Adenovirus expressing HA-tagged Akt with the c-src myristolyation sequence fused in frame to the N terminus (Hyper active Akt), HA-tagged Akt dominant mutant (T308A, S473A) was purchased from Vector Biolabs (Eagleville, PA). Control or IPF fibroblasts were infected with adenovirus expressing PTEN, Akt or empty vector on tissue culture plates. 2×10^5^ control or IPF fibroblasts per ml of DMEM medium were infected with 1×10^6^ PFU of each adenovirus before attachment to polymerized collagen. Protein expression was quantified using LabWorks Image acquisition and analysis software version 4.6 (UVP BioImage systems, CA). Each band was normalized to GAPDH and presented.

### Immunofluorescence

For the immunofluorescence assay, IPF fibroblasts cultured on polymerized collagen were fixed for 10 minutes in cold methanol, rinsed with PBS, and blocked in 5% Donkey serum in PBS buffer for 30 minutes at room temperature (RT). Cells were then incubated with LC3-2 primary antibody (catalog No. 3868, Cell Signaling Technologies) overnight at 4°C, washed 3 times with PBS, and incubated in secondary antibody Donkey anti-Rabbit Cy 3 (1∶500, Jackson ImmunoResearch, West Grove, PA) diluted in PBS for 45 min at RT. After rinsing with PBS, slides were incubated in DAPI at RT for 20 min in the dark, washed, and mounted with Prolong Gold antifade reagent (Invitrogen, Eugene, Oregon). Image analysis was performed on a Zeiss Axiovert 200, and processed using Axiovision Version 4.7 software. In a secondary experiment performed with the exact protocol stated above, Rabbit mAB- IgG XP Isotype Control (catalog No. 3900, Cell Signaling Technologies) was used in place of the primary antibody. Image analysis was performed on an Olympus FluoView 1000 IX2 and processed using FV-10 ASW 2.1 software. Fluorescent images were quantified using a public domain Java image processing program, ImageJ obtained from http://rsbweb.nih.gov/ij.

### Immunohistochemistry

Patient specimens were fixed overnight with paraformaldehyde (4%) at RT. The specimens were then rinsed in 6% sucrose/0.1 M Dulbeccos PBS (DPBS) at RT, infiltrated with increasing concentrations of sucrose/DPBS with rotation at 4°C over a 72 h period, and cryoprotected first with 20% sucrose/DPBS overnight, then with a 1∶1 solution of 20% sucrose in DPBS/O.C.T (Sakura Finetek, Torrance, CA) and rotated overnight at 4°C. After cryoembedding in O.C.T., 4 µ sections were cut and placed on silane-coated slides using a Leica 1950 Cryostat. Cryosections were fixed in 100% methanol at −20°C for 10 minutes, air dried and then quenched with 0.3% Hydrogen peroxide in methanol for 15 minutes at RT, and again air dried. They were then rinsed in PBS, placed in a steamer for 10 min with Citrate Buffer for antigen retrieval, and blocked for 30 min with normal serum determined by the host species of the antibody for blocking nonspecific binding of secondary antibodies. Endogenous Avidin and Biotin binding sites were blocked by sequential incubation with Avidin/Biotin Blocking Kit (Vector Laboratories, Burlingame, CA). The sections were then incubated overnight at 4°C in their respected concentrations for each antibody, p-mTOR (1∶100, catalog No. 2976, Cell Signaling Technologies, MA), LC3-2 (1∶150, catalog No. 3868, Cell Signaling Technologies, MA). For detection of the primary antibody, the LSAB Method was used. The sections were first rinsed with PBS, then placed in a species specific biotinylated secondary antibody for 30 min (1∶500), and transferred to the HRP-Streptavidin (Vector Laboratories, Burlingame, CA) conjugate solution for 30 min. The enzyme was then visualized with the application of the chromagen substrate 3, 3′ Diaminobenzene and counterstained with Hematoxylin. Control specimens were processed with the identical protocol minus the primary antibody to produce the negative controls.

### Statistical Analysis

Protein expressions are presented as a box-and-whisker plot showing the lowest expression, lower quartile, median, upper quartile and the highest expression using SPSS v.19. Two dimensional column graphs were prepared using Excel. Data are expressed as the means ± S.D. Group comparisons between IPF and control were conducted using Student's t-tests. Significance level was set at p<0.05 (two-tailed).

## Results

### Autophagy was suppressed in IPF fibroblasts following attachment to polymerized collagen

**Figure 1:**
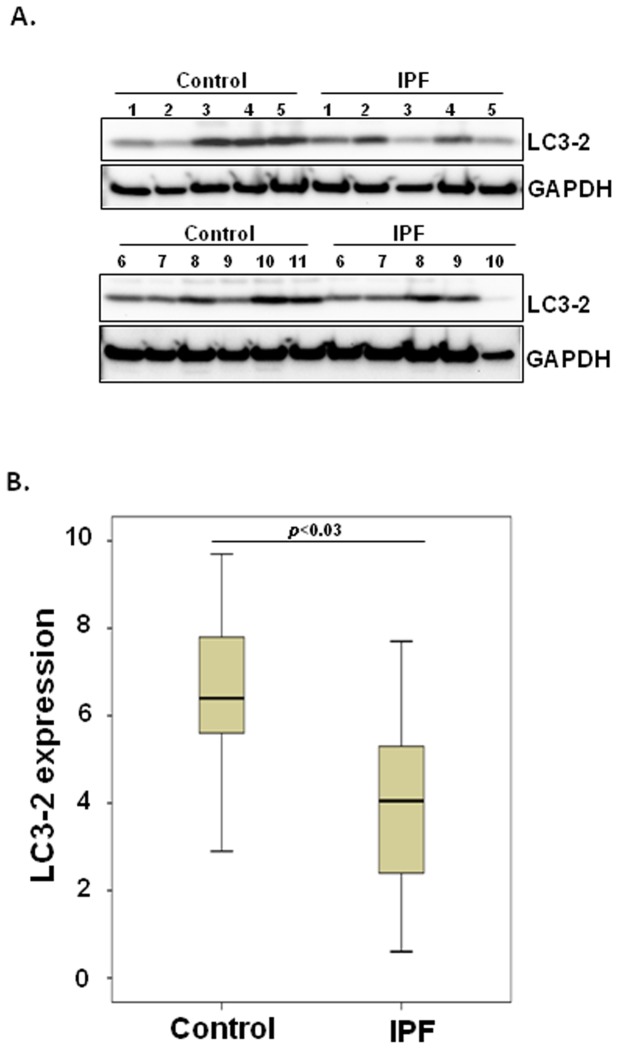
LC3-2 expression is low in IPF fibroblast attachment to polymerized collagen. A) 2×10^5^ serum starved control (n = 11) and IPF fibroblasts (n = 10) were attached to type I polymerized collagen (2 mg/ml) in serum free medium and LC3-2 and GAPDH expression was measured at 24 h. B) Shown is a box-and-whisker plot that LC3-2 is normalized to GAPDH in control and IPF fibroblasts as described in the Materials and Methods. *p*<0.03 IPF versus control fibroblasts. All control and IPF fibroblasts were randomly selected for this assay.

Prior study showed that LC3-2 protein level is low in IPF lung tissue [41]. This study indicated a possibility that IPF fibroblasts have aberrantly low autophagy on polymerized collagen, which may be responsible for the promotion of fibrogenesis in IPF. To test this, we first measured LC3-2 expression, which is widely used to monitor autophagy in control (n = 11) and IPF fibroblasts (n = 10) cultured on polymerized collagen. Although there is a biological variability in LC3-2 expression in control and IPF fibroblasts cultured on collagen, overall, LC3-2 expression was lower in IPF fibroblasts compared with that of control fibroblasts (Fig. 1A&B). We further examined LC3-2 expression in each control and IPF fibroblast line to validate their expression patterns. We found that most of the IPF fibroblast lines have relatively low LC3-2 expression while the majority of control fibroblast lines have enhanced LC3-2 levels (Figure S1A & S1B). Taken together, these data show that autophagy induction is aberrantly suppressed when IPF fibroblasts interact with collagen rich matrix.

### IPF fibroblasts maintain enhanced mTOR kinase activity due to an aberrant PTEN/Akt axis on polymerized collagen

**Figure 2:**
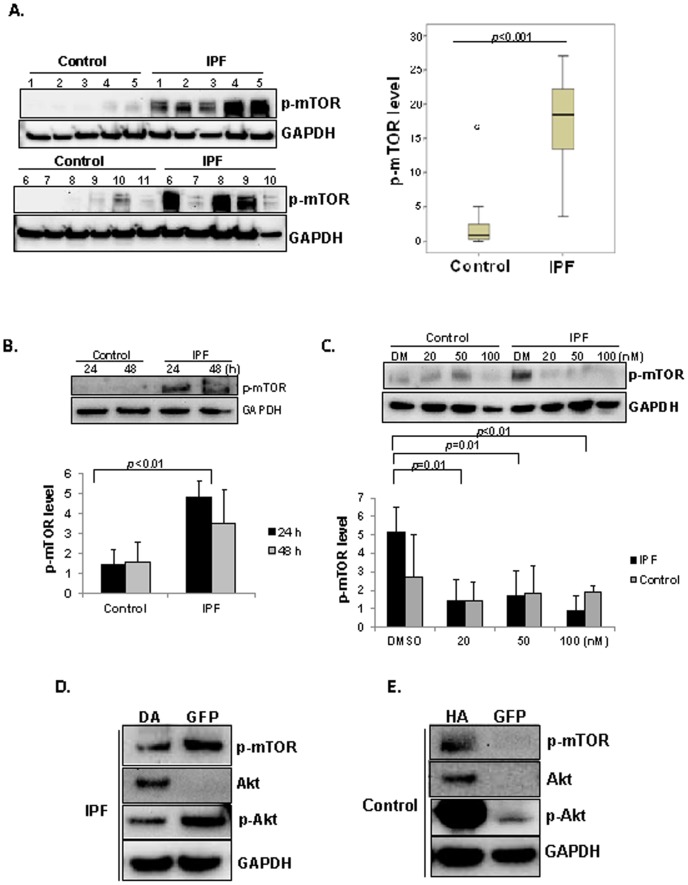
mTOR kinase activity remains high when IPF fibroblasts are cultured on collagen matrix. A) Left, 2×10^5^ serum starved control lung (n = 11) and IPF fibroblasts (n = 10) were cultured on polymerized collagen in serum free medium for 24 h and ser 2448 p-mTOR (p-mTOR) levels were measured by Western analysis. Shown is a Western analysis for p-mTOR levels in all IPF and control fibroblasts grown in serum free medium on polymerized collagen. GAPDH was used as a loading control. Right, Shown is a box-and-whisker plot of p-mTOR protein expression in 11 control and 10 IPF fibroblasts normalized to GAPDH on collagen matrix. *p*<0.001 IPF versus control fibroblasts. B) Upper, randomly selected 2×10^5^ of 3 control or of 3 IPF fibroblasts were cultured on polymerized collagen in serum free medium as a function of time and ser 2448 p-mTOR levels were measured. GAPDH was used as a loading control. Lower, shown is the p-mTOR/GAPDH protein expression ratio in randomly selected control and IPF fibroblasts as a function of time. *p*<0.01 IPF versus control fibroblasts at 24 h. C) Upper, control and IPF fibroblasts (n = 5 each) were cultured in the presence of various doses of rapamycin on collagen gel for 24 h and p-mTOR level was then measured. Shown is the representative p-mTOR protein level in control and IPF fibroblasts on polymerized collagen. GAPDH level was used as a loading control. Lower, p-mTOR/GAPDH expression ratio in control (n = 5) and IPF fibroblasts (n = 5) in the presence of various doses of rapamycin on collagen are shown. *p* = 0.01 versus DMSO control. *p*<0.01 versus DMSO control as indicated. Cells were randomly selected for this assay. D) IPF fibroblasts infected with adenovirus expressing dominant negative Akt (DA) or empty vector GFP were cultured on collagen gel for 24 h in serum free medium and p-mTOR, Akt, phosphorylated Akt and GAPDH levels were measured. IPF fibroblasts expressing enhanced p-mTOR level was selected to examine whether Akt inhibition suppresses p-mTOR level (lane 5 in upper panel of IPF in Fig 2A). This assay was repeated twice. E) Control fibroblasts infected with adenovirus expressing hyperactive Akt (HA) or empty vector (GFP) were cultured on collagen for 24 h in serum free medium and p-mTOR, total Akt, phosphorylated Akt and GAPDH levels were measured. Control fibroblasts expressing low p-mTOR level were selected to examine whether hyperactive Ak increases mTOR kinase activity (lane 3 in upper panel of control fibroblasts in Fig 2A). This assay was repeated twice.

IPF fibroblasts elude the proliferation inhibitory property of polymerized collagen matrix via elevated levels of Akt due to PTEN suppression [22], [23]. Since autophagic function is predominantly regulated by Akt/mTOR kinases, this finding suggests that autophagic activity remains low when IPF fibroblasts are attached to polymerized collagen as a result of abnormally enhanced mTOR activity, a concept we previously published [42]. Therefore, we next hypothesized that IPF fibroblasts have enhanced mTOR kinase activity due to the aberrant PTEN/Akt axis thereby inhibiting autophagic activity on collagen. To test this hypothesis, we first measured mTOR kinase activity using ser 2485 phosphorylated mTOR level as a surrogate marker from randomly selected 11 control and 10 IPF fibroblasts. Overall, the phosphorylated mTOR protein level was significantly higher in IPF fibroblasts compared with that of control fibroblasts cultured on collagen (Fig. 2A left and right). Likewise, when we measured p-mTOR expression in each control and IPF cell line in Fig. 1A & 2A, p-mTOR expression was highly up-regulated in most individual IPF cell lines compared to control cells (Figure S1A & 1B). Therefore, taken together, our results showed that there is a clear distinction between control and IPF fibroblasts in that p-mTOR and LC3-2 are inversely related to each other, and autophagy is abnormally altered in IPF fibroblasts in response to collagen matrix. We next examined mTOR kinase activity in IPF and control fibroblasts as a function of time. mTOR kinase activity was high and relatively unaltered in response to IPF fibroblast attachment to collagen (Fig. 2B upper lanes 3&4 and lower panel). In contrast, phosphorylation of mTOR was barely detectable in control fibroblasts as a function of time (lanes 1&2). These data suggest that when IPF fibroblasts attach to polymerized collagen, the activity of mTOR kinase is up-regulated and remains high. To confirm this finding that mTOR kinase is abnormally high in IPF fibroblasts on collagen, we next treated control and IPF fibroblasts with various doses of mTOR kinase inhibitor rapamycin and measured mTOR activity on collagen matrix. When control fibroblasts were cultured on collagen in the presence of various doses of rapamycin, p-mTOR level was relatively unaffected compared to that of DMSO treated cells (Fig. 2C, lanes 1 to 4, upper and lower panel). In contrast, high p-mTOR level was clearly abolished when IPF fibroblasts were cultured on polymerized collagen in the presence of rapamycin (lanes 6 to 8). Collectively, these data showed that unlike control fibroblasts, IPF fibroblasts exhibit enhanced mTOR kinase activity and rapamycin significantly inhibits mTOR kinase function.

mTOR is a down-stream kinase of Akt [30] and Akt activity is abnormally high in IPF fibroblasts on collagen [19], [20], [35]. To confirm that high mTOR kinase activity is due to aberrant Akt activity in IPF fibroblasts, IPF fibroblasts expressing enhanced p-mTOR were selected, and Akt function was inhibited by dominant negative Akt. The p-mTOR level was low when IPF cells were infected with adenovirus expressing dominant negative Akt (Fig. 2D). Since Akt activity was low in response to control fibroblast attachment to collagen matrix, Akt was over-expressed in control fibroblasts expressing low p-mTOR, and mTOR kinase activity was also measured. Phosphorylated mTOR level was significantly increased when hyperactive Akt was over-expressed compared with that of empty GFP infected control cells (Fig. 2E). Taken together, these data further demonstrated that the alteration of mTOR kinase activity is due to aberrant Akt function.

### Aberrantly high mTOR kinase suppresses autophagic activity in IPF fibroblasts on polymerized collagen

**Figure 3:**
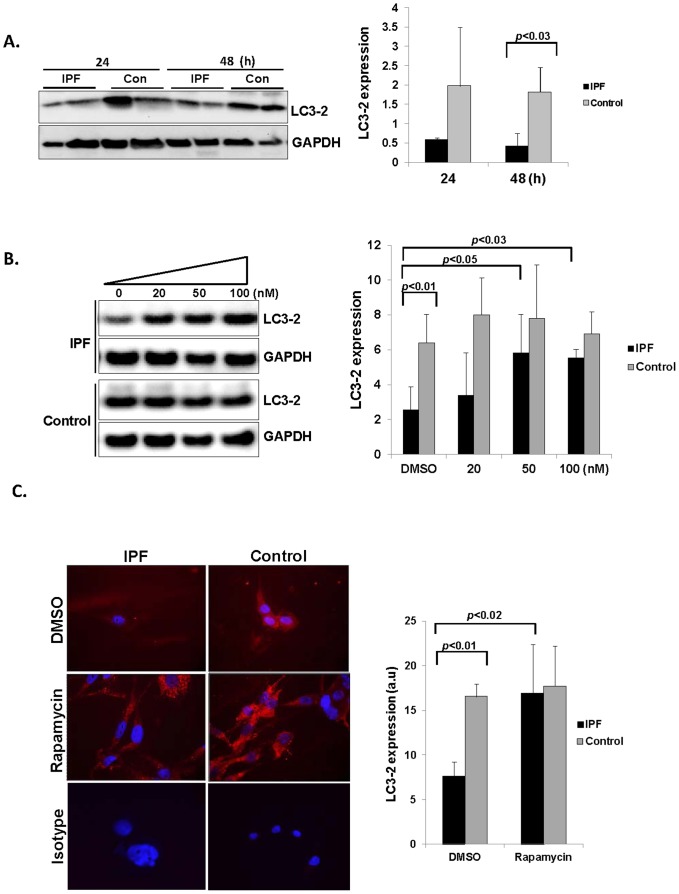
LC3-2 expression was up-regulated in IPF fibroblasts in the presence of rapamycin on collagen. A) Left, 2×10^5^ serum starved 3 IPF and 3 control fibroblasts were attached to polymerized collagen as a function of time and LC3-2 and GAPDH was measured. Shown is a representative Western analysis IPF and control cells grown on polymerized collagen at 24 and 48 h. IPF and control fibroblasts were randomly selected for this assay. Right, shown is LC3-2 expression in 3 control and 3 IPF fibroblasts normalized to GAPDH. *p*<0.03 versus control at 48 h. B) Left, equal number of control and IPF fibroblasts were cultured on polymerized collagen in serum free medium in the presence of various doses of rapamycin as indicated, and LC3-2 expression was measured at 24 h. GAPDH was used as a loading control. Shown is a representative Western analysis for LC3-2 expression. Right, shown is the LC3-2/GAPDH protein expression ratio in 4 control and 4 IPF fibroblasts in the presence of various doses of rapamycin. *p*<0.03 versus DMSO, *p*<0.05 versus DMSO, *p*<0.01 versus control fibroblasts as indicated. Note that LC3-2 expression was highly up-regulated when IPF fibroblasts were cultured in the presence of rapamycin while LC3-2 expression was not significantly altered when control fibroblasts were treated with various doses of rapamycin. These control and IPF fibroblasts were randomly selected. C) Left, LC3-2 (red) expression was measured in randomly selected IPF and control fibroblasts cultured on cover slips coated with polymerized collagen in the presence of 100 nM of rapamycin using immunofluorescent microscopic analysis. Note that LC3-2 expression was high when rapamycin was used in IPF fibroblasts. DAPI (blue) was used as a nuclear counterstain. Rabbit IgG was used as a control and images were taken by an immunofluorescent microscope. Data are representative of 2 independent experiments. Original magnification, 63X, rabbit IgG isotype 60X. DMSO: DMSO control. Right, the quantification of fluorescence of autophagy in IPF and control fibroblasts on collagen. The fluorescence intensities of autophagy in IPF and control fibroblasts were quantified as described in the Materials and Methods. Shown is the relative fluorescence intensities of LC3-2 in two randomly selected IPF and control fibroblasts in the presence of DMSO or rapamycin. Y axis scale is presented in arbitrary units (a.u). *p*<0.01 versus control, *p*<0.02 versus DMSO as indicated.

We next examined whether high mTOR kinase is responsible for the inhibition of autophagosome expression. Since p-mTOR expression remained high when IPF fibroblasts were attached to polymerized collagen at various time points, we first measured LC3-2 expression in control and IPF fibroblasts on collagen as a function of time. LC3-2 expression was low and relatively unaltered when IPF fibroblasts were cultured on collagen (Fig. 3A, left and right). In contrast, control fibroblasts expressed high LC3-2 protein and remained high as a function of time. These results further support our results that autophagic activity is abnormally suppressed upon IPF fibroblast's attachment to collagen. To test whether high mTOR activity is responsible for LC3-2 expression, we next measured LC3-2 expression in control and IPF fibroblasts in the presence of various doses of rapamycin on collagen matrix. When IPF fibroblasts were treated with 20 to 100 nM of rapamycin, LC3-2 expression was ∼2.5 fold increased (Fig. 3B, left and right panels). In contrast, LC3-2 expression was high in control fibroblasts and remained relatively unaltered in the presence of various doses of rapamycin. To confirm these findings, control and IPF fibroblasts were treated with rapamycin, and LC3-2 expression was examined by immunofluorescence. Like the Western analysis in Fig 3A, LC3-2 expression was low in the absence of rapamycin (DMSO treated cells) while LC3-2 expression was significantly increased when IPF fibroblasts were treated with rapamycin (Fig. 3C left and right). In contrast, LC3-2 expression was high in the presence of DMSO and slightly increased when control fibroblasts were treated with rapamycin (Fig. 3C, left and right). Collectively, these data demonstrate that the inhibition of high mTOR kinase activity by rapamycin significantly increases autophagosome formation in IPF fibroblasts on polymerized collagen, while mTOR inhibition has relatively minor effect on autophagic activity in control fibroblasts due to their inherently low mTOR activity.

### The PTEN/Akt/mTOR axis regulates autophagosome formation

**Figure 4:**
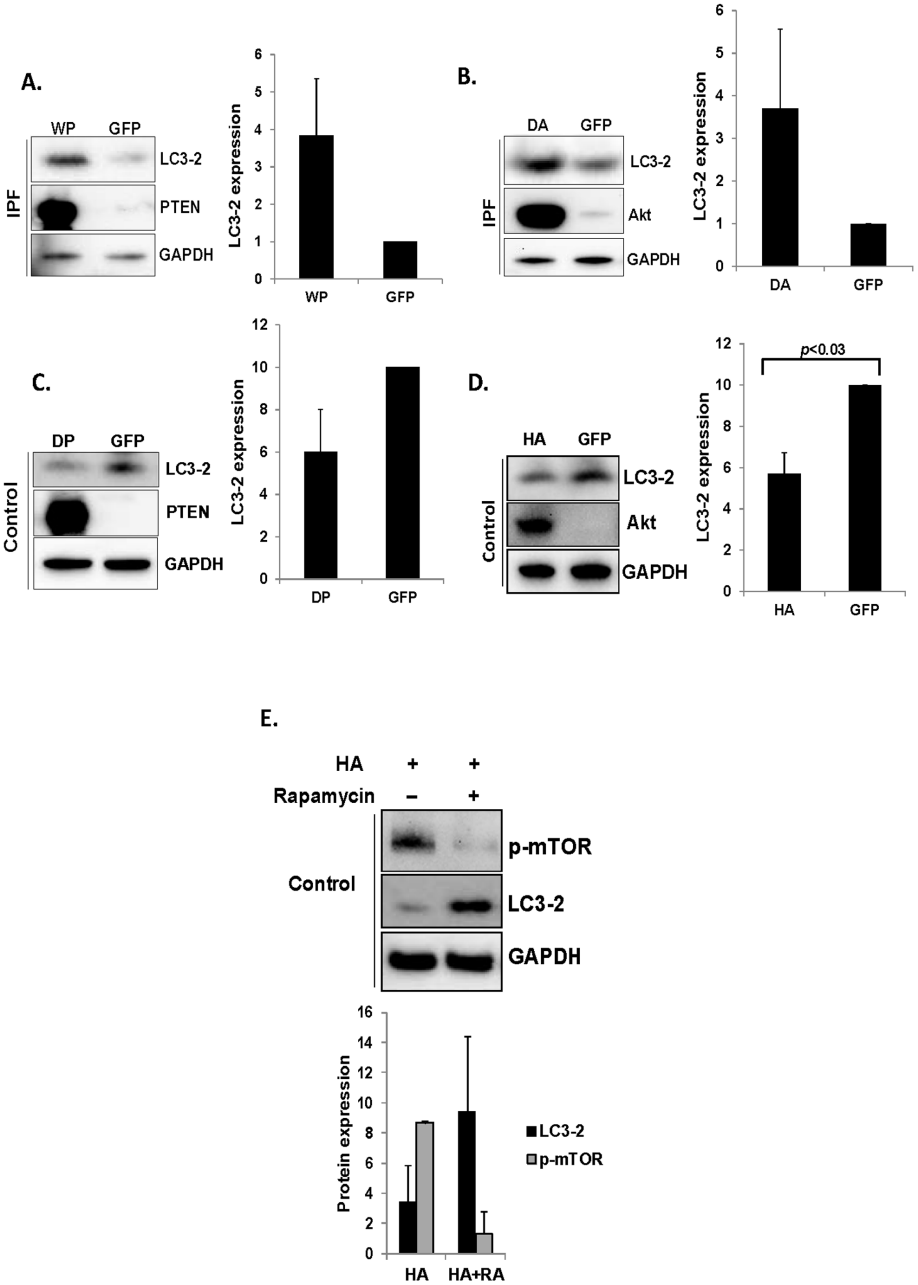
PTEN/Akt/mTOR axis regulates autophagosome formation. A) Left, 2×10^5^ IPF fibroblasts expressing wild type PTEN (WP) or empty vector (GFP) were cultured on polymerized collagen matrix for 24 h in serum free medium and LC3-2, PTEN and GAPDH expression were measured. Right, LC3-2/GAPDH expression in IPF fibroblasts expressing wild type PTEN or GFP on collagen was measured using densitometry. B) IPF fibroblasts expressing dominant negative Akt (DA), or empty vector (GFP) were cultured on collagen matrix for 24 h and LC3-2, Akt and GAPDH expression was measured. Right, LC3-2/GAPDH expression in IPF fibroblasts expressing dominant negative Akt or GFP on collagen was measured using densitometry. C) Control fibroblasts expressing mutant PTEN (DP) or empty vector (GFP) were cultured on collagen matrix for 24 h in serum free medium and LC3-2, PTEN, and GAPDH expression was measured. Right, LC3-2/GAPDH expression in control fibroblasts expressing mutant PTEN or GFP on collagen was measured using densitometry. D) Control fibroblasts expressing hyperactive Akt (HA) or empty vector (GFP) were cultured on collagen matrix for 24 h and LC3-2, Akt, and GAPDH expression was measured. Right, LC3-2/GAPDH expression in control fibroblasts expressing hyperactive Akt or GFP on collagen was measured using densitometry. *p*<0.03 versus GFP. E) Upper, control fibroblasts infected with adenovirus expressing hyperactive Akt (HA) were cultured on collagen in the presence or absence of 100 nM of rapamycin for 24 h and p-mTOR, LC3-2, and GAPDH expression were measured. Lower, LC3-2/GAPDH, p-mTOR/GAPDH expression ratios were measured using densitometry. Shown are representative Western analysis for LC3-2 expression in IPF or control fibroblasts expressing LC3-2 or p-mTOR on collagen. All experiments were obtained from three independent assays. These assays were performed with IPF fibroblasts shown in lanes 1, 4 and 5 in an upper panel in Fig 1A, and with control fibroblasts shown in lanes 3 and 5 in an upper panel in Fig 1A.

Our data demonstrate that the inhibition of inappropriately high Akt decreases mTOR kinase activity in IPF fibroblasts (Fig. 2D). Our prior studies have demonstrated that Akt activity is up- regulated as a result of PTEN suppression in IPF fibroblasts on collagen matrix [20], [21]. Therefore, we next measured LC3-2 expression in IPF and control fibroblasts to verify the role of PTEN and Akt on autophagosome regulation. PTEN over-expression increased approximately 4 fold the LC3-2 expression in IPF fibroblasts (Fig. 4A). Furthermore when Akt was inhibited by dominant negative Akt in IPF fibroblasts, LC3-2 expression was increased more than 3 fold (Fig. 4B). In contrast, when PTEN was suppressed by mutant PTEN in control fibroblasts, LC3-2 expression was low compared with that expressing empty vector GFP (Fig. 4C). Likewise, LC3-2 expression level was low when hyperactive Akt was expressed in control fibroblasts on collagen (Fig. 4D). These data demonstrate that unlike control fibroblasts, when IPF fibroblasts attach to polymerized collagen, enhanced mTOR kinase inhibits autophagic activity due to the aberrant PTEN/Akt axis. To examine whether Akt activity is essential in regulating LC3-2 via mTOR kinase, control fibroblasts were infected with adenovirus expressing hyperactive Akt or empty vector in the presence or absence of rapamycin and LC3-2 expression was measured. When hyperactive Akt was over-expressed in the absence of rapamycin, LC3-2 expression was very low (Fig. 4E, lane 1 & lower panel). However, LC3-2 protein level remained high when hyperactive Akt was over-expressed in the presence of rapamycin (lane 2). These data further support our findings that LC3-2 expression is regulated via the Akt/mTOR-dependent pathway, and unlike control fibroblasts, Akt and mTOR activities are high in response to IPF fibroblast attachment to polymerized collagen, which subsequently down-regulates autophagosome formation.

### IPF fibroblasts are desensitized to collagen rich matrix induced cell death in the presence of 3MA or CQ

**Figure 5:**
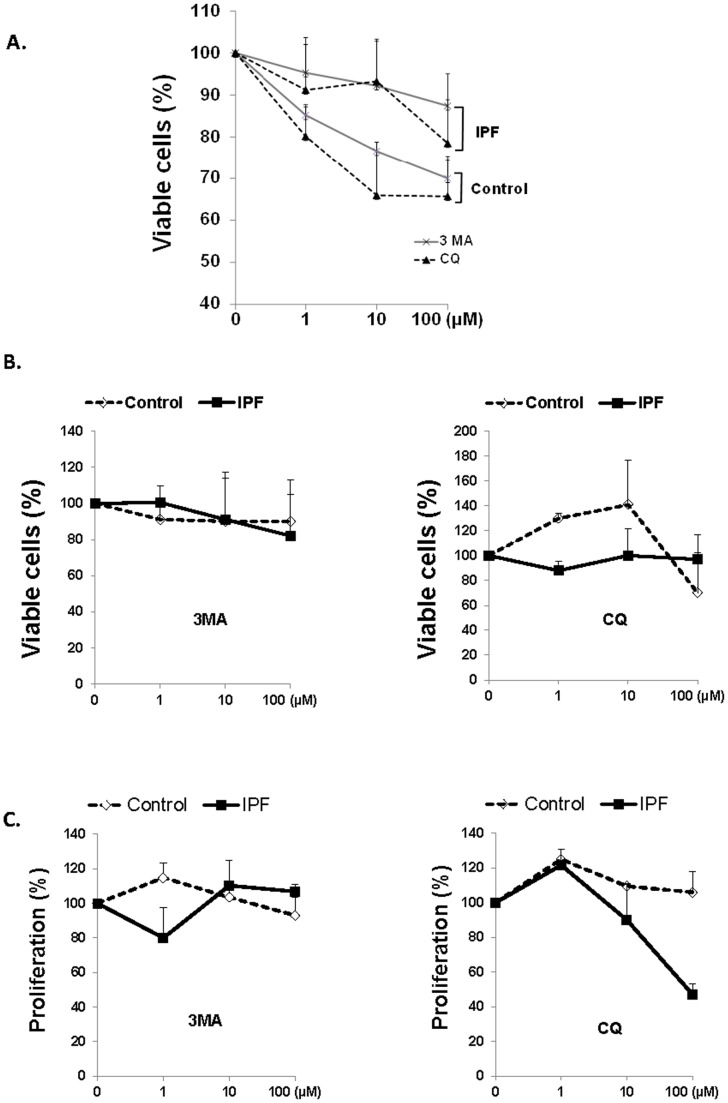
IPF fibroblasts are desensitized to collagen rich matrix induced cell death in the presence of 3MA or CQ. A) 3×10^4^ control or IPF fibroblasts were cultured on polymerized collagen for 24 h in serum free medium in the presence of various doses of 3-methyladenine (3MA) or chloroquine (CQ), and cell viability was measured as described in the Materials and Methods. Shown is the % decrease in control and IPF fibroblasts in the presence of autophagic inhibitors on polymerized collagen (the viable IPF and control fibroblasts on collagen matrix in the absence of inhibitors (0 µM) are considered as 100% viable cells). B) 3×10^4^ control or IPF fibroblasts were cultured on 96 well tissue culture plates in the absence of polymerized collagen in serum free medium, and viable cells were measured in the presence of various doses of 3-methyl adenine (3MA, left panel) or chloroquine (CQ, right panel) for 24 h. Shown is the % decrease in viability in control and IPF fibroblasts in the presence of 3MA or CQ. All assays were performed in triplicate. C) 3×10^4^ control or IPF fibroblasts were cultured on polymerized collagen in serum free medium, and fibroblast proliferation was measured in the presence of 3MA (left panel) or CQ (right panel) for 24 h as described in the Materials and Methods. Shown is the % decrease in proliferation in control and IPF fibroblasts in the presence of 3MA or CQ. All assays were performed in three separate experiments to measure fibroblast viability or their proliferation using IPF cells in lane 5 and control fibroblasts in lane 3 in an upper panel in Fig 1A.

Prior studies showed that cells utilized autophagy as a temporary survival mechanism under a stress inducing environment [8], [13], [14], [45]. Our data show that autophagic activity is abnormally low in IPF fibroblasts in response to polymerized collagen, which suggests that IPF fibroblasts do not sense polymerized collagen as a stress-inducing condition. Since deregulation of autophagic activity has been linked to the alteration of cell death [6]–[8], we next examined whether abnormal autophagic activity is responsible for the suppression of IPF fibroblast cell death. To test this, control and IPF fibroblasts were grown on polymerized collagen in the presence of various doses of autophagy inhibitors, 3-methyladenine (3MA) or chloroquine (CQ), and cell viability was measured. If autophagy protects IPF cells from collagen induced cell death, the inhibition of autophagy can increase cell death. Viability of IPF fibroblasts was moderately decreased in the presence of 1 to 100 µM of 3MA or CQ (∼10% and ∼20% decrease in cell viability when 100 µM of 3MA or CQ used, respectively, Fig. 5A). In contrast, there was significant decrease in viable cells when control fibroblasts were treated with 3MA or CQ (∼30% and ∼35% decrease in cell viability when 100 µM of 3MA or CQ was used, respectively). These data show that IPF fibroblasts with low autophagy activity are less susceptible to polymerized collagen induced cell death when autophagic activity is inhibited due to pre-existing low autophagic activity. In contrast, control fibroblasts with enhanced autophagic activity become highly sensitized to polymerized collagen induced cell death in the presence of autophagy inhibitors, which further suggests that abnormal alteration of autophagy is linked to fibroblast viability. In order to verify whether autophagy inhibitors have an intrinsic toxic effect on viability of fibroblasts, IPF and control fibroblasts were treated with various doses of 3MA or CQ on tissue culture plates in the absence of polymerized collagen, and viable cells were measured. Unlike the results in 5A, when IPF and control cells were cultured with various doses of 3MA, no significant changes were found in viable cells (Fig. 5B, left panel). The treatment of IPF fibroblasts with various doses of CQ also did not alter their viability while only the highest dose of CQ promoted control fibroblast cell death (∼30%, Fig 5B, right panel). Since the treatment of IPF and control fibroblasts with various doses of 3MA or CQ predominantly increased control fibroblast cell death on collagen matrix (∼22% and ∼35% decrease in cell viability in control fibroblasts while ∼5% decrease in viable IPF fibroblasts in the presence of 10 µM of 3MA or CQ, Fig 5A), these data strongly suggest that except for the treatment of control fibroblasts with the highest dose of CQ, the toxic effect of 3MA or CQ on IPF and control cells are absent or minimal.

We previously found that IPF fibroblasts are highly proliferative on polymerized collagen [22], [23]. Since IPF fibroblasts are more resistant to cell death when autophagic activity was inhibited on collagen matrix due to pre-existing low autophagic activity, we next sought to examine whether high proliferation of IPF fibroblasts is the main reason for the enhanced viability in IPF fibroblasts. To test this possibility, we examined IPF and control fibroblast proliferation on polymerized collagen in the presence of various doses of 3MA or CQ. The treatment of 3MA did not alter overall IPF and control fibroblast proliferation (Fig. 5C, left panel). However, unlike control fibroblasts, IPF fibroblast proliferation was significantly reduced in the presence of 10 and 100 µM of CQ (∼12, 53%, respectively, Fig. 5C, right panel). Taken together, these data showed that enhanced viability in IPF fibroblasts in the presence of autophagy inhibitors is not due to high proliferation but rather due to a desensitized IPF fibroblast property in response to collagen.

### IPF fibroblasts are sensitized to collagen rich matrix induced cell death via the PTEN/Akt axis in the presence of 3MA or CQ

**Figure 6:**
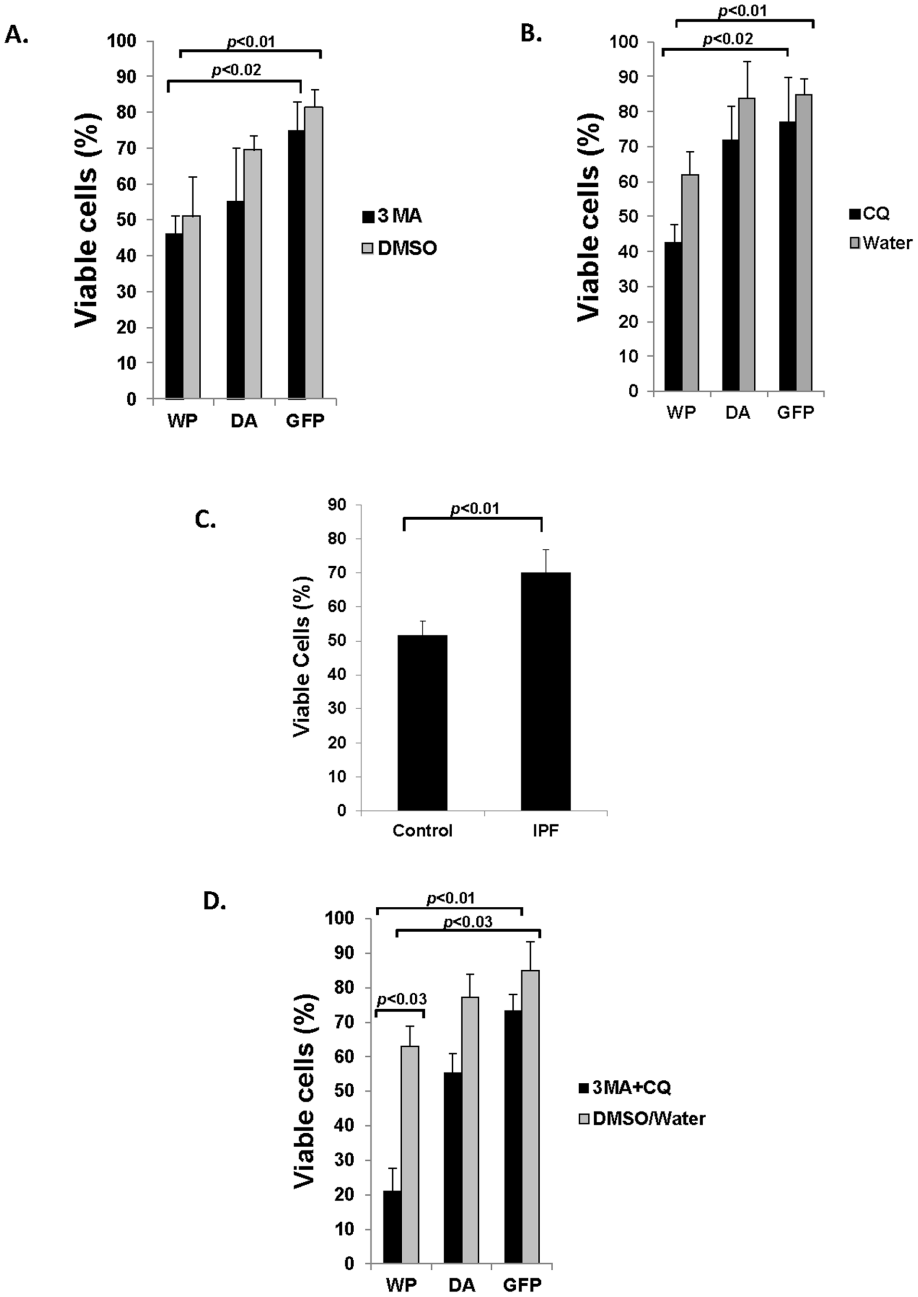
Suppression of autophagic activity in IPF fibroblasts over-expressing PTEN or dominant negative Akt increases IPF fibroblast cell death on collagen. A) 3×10^4^ IPF fibroblasts infected with adenovirus expressing wild type PTEN (WP), dominant negative Akt (DA) or empty vector (GFP) were cultured on polymerized collagen in the presence of 100 µM of 3MA or DMSO in serum free medium and viable cells were measured at 24 h. *p*<0.01 versus GFP, *p*<0.02 versus GFP. B) IPF fibroblasts infected with adenovirus expressing wild type PTEN (WP), dominant negative Akt (DA) or empty vector (GFP) were cultured on polymerized collagen in the presence of 100 µM of CQ or water in serum free medium and viable cells were measured at 24 h. *p*<0.01 versus GFP, *p*<0.02 versus GFP. C) 3x10^4^ control or IPF fibroblasts grown on 96 well plates coated with polymerized collagen were treated with 100 µM of 3 methyl adenine (MA) and chloroquine (CQ) together, and viable cells were measured at 24 h in serum free medium. Shown is the % change in viable control or IPF fibroblasts treated with 3MA and CQ on collagen matrix. *p*<0.01 versus control fibroblasts. D) IPF fibroblasts infected with adenovirus expressing wild type PTEN (WP), dominant negative Akt (DA) or empty vector (GFP) cultured on polymerized collagen matrix were treated with both 100 µM of 3MA and CQ together (3MA+CQ), and viable cells were measured at 24 h. *p*<0.01 versus GFP, *p*<0.03 versus GFP, *p*<0.03 versus DMSO/water as indicated. All assays were performed in triplicate using IPF cells shown in lane 5 or control fibroblasts in lane 3 in an upper panel in Fig 1A.

Our data shows that LC3-2 expression is low in IPF fibroblasts cultured on collagen but expression is up-regulated by PTEN over-expression or Akt inhibition (Fig. 4A&B). This finding suggests that IPF fibroblasts do not sense polymerized collagen as an unfavorable environment due to the altered PTEN/Akt axis and this low autophagy enables IPF fibroblasts to be desensitized to collagen matrix induced cell death, thereby efficiently maintaining their viable phenotype. To verify this, we next examined whether the inhibition of high autophagic activity induced by the forced expression of PTEN or dominant negative Akt can promote IPF fibroblast cell death on collagen. Since intrinsic toxicity was absent or very low in IPF fibroblasts treated with various doses (1 to 100 µM) of 3MA or CQ, we next measured cell viability in IPF fibroblasts expressing PTEN or dominant negative Akt in the presence of 10 or 100 µM of 3MA or CQ. The treatment of IPF fibroblasts with either 10 µM of 3MA or CQ marginally decreased their viability compared with DMSO or water treated cells (Figure S2A & S2B). To verify whether the higher dose of autophagic inhibitors further decreases IPF cell viability on collagen, IPF fibroblasts expressing PTEN or dominant negative Akt were cultured on collagen in the presence of 100 µM of 3MA or CQ, and cell viability was also measured. Akt inhibition with 3MA marginally decreased viable cells in comparison to cells expressing dominant Akt in the absence of 3MA (∼55% vs ∼70% viable cells, respectively, Fig. 6A). Likewise, when IPF cells expressing PTEN (WP) were treated with 3MA, viable cells were slightly decreased (∼45% vs ∼52% viable cells, respectively). However, when IPF fibroblasts expressing wild type PTEN were treated with CQ, there was an apparent decrease in viable cells in IPF fibroblasts on collagen matrix compared with cells expressing wild type PTEN in the absence of CQ (∼43% vs ∼62% viable cells, Fig. 6B). Likewise, Akt inhibition using dominant negative Akt also marginally decreased viable cells compared with IPF fibroblasts not treated with CQ (72% vs 84% viable cells, respectively). Collectively these data showed that the alteration of the PTEN/Akt axis enhances the autophagic process and the subsequent inhibition of autophagic activity sensitizes IPF fibroblasts to polymerized collagen induced cell death.

Our data strongly suggests that the inhibition of autophagy alone does not significantly increase IPF cell death on polymerized collagen matrix due to pre-existing low autophagic activity. Research has shown that 3MA inhibits the early phase of the autophagic process while CQ disrupts the function of lysosomes, thus acting at a later stage [39], [40]. Therefore, we next utilized both 3MA and CQ together to inhibit various stages of the autophagic process in control and IPF fibroblasts and measured viable cells. Approximately 70% of IPF fibroblasts were viable in the presence of both 3MA and CQ (Fig 6C). However, only ∼50% of control cells were viable when both 3MA and CQ was used. These findings further supported our result that IPF fibroblasts become less susceptible to polymerized collagen induced cell death due to inherently low autophagic activity. To confirm this finding, we next examined whether PTEN or Akt modulation in the presence of both 3MA and CQ together can increase IPF fibroblast cell death. When 10 µM of 3MA and CQ was used in IPF fibroblasts expressing PTEN, there was a significant decrease in cell viability compared with DMSO/water treated cells (∼35% decrease in viable cells, Figure S2C). Furthermore, when IPF cells expressing PTEN were treated with 100 µM of both 3MA and CQ together (Fig. 6), there was substantial decrease (∼42%) in viable cells (Fig. 6D). Likewise, when IPF cells expressing dominant negative Akt were cultured on collagen in the presence of autophagy inhibitors, viable cells were also decreased (∼20%). Collectively, these data demonstrate that when IPF fibroblasts attach to polymerized collagen, the altered PTEN/Akt pathway inhibits autophagy activity, which subsequently desensitizes IPF fibroblasts from polymerized collagen-induced cell death. However, IPF fibroblasts can become re-sensitized to polymerized collagen induced cell death by the forced activation of PTEN or dominant negative Akt in the presence of autophagy inhibitors.

### mTOR activity is high while LC3-2 is low in cells within the fibroblastic foci of patients with IPF

**Figure 7:**
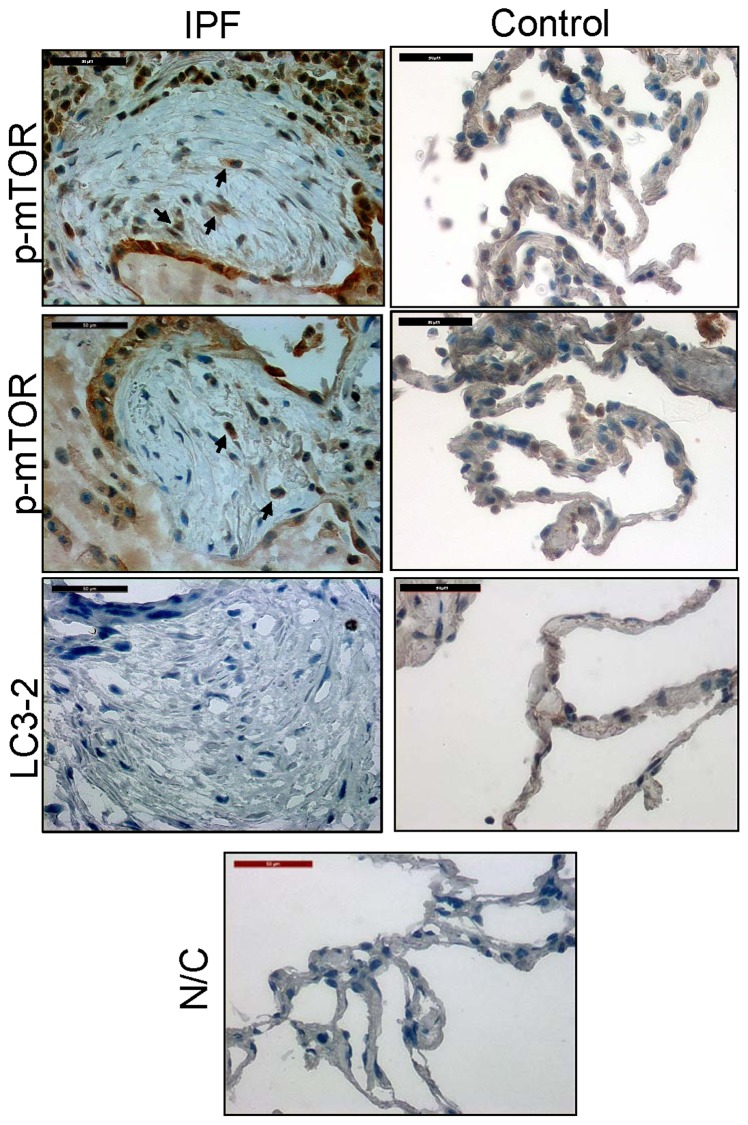
mTOR activity is high while LC3-2 is low in cells within the fibroblastic foci of patients with IPF. IHC was performed with lung tissues from IPF patients or from histologically normal lungs (both n = 2) using p-mTOR and LC3-2 antibodies. These specimens were counterstained with hematoxylin. Shown is p-mTOR and LC3-2 expression in cells within the fibroblastic foci of IPF patients (left panels) or in normal lung alveoli tissue specimens (right panels). Lower panel, N/C is a negative control without IHC specific primary antibody. Arrows indicate p-mTOR positive cells. Note that p-mTOR level is high while LC3-2 expression is low in cells within the fibroblastic foci from IPF patients.

Since autophagic activity is low in IPF fibroblasts cultured on polymerized collagen, we next sought to examine whether our *in vitro* results can be also found in myofibroblasts within the fibroblastic foci of IPF patient specimens. We previously demonstrated that cells within the fibroblastic foci are α smooth muscle actin positive myofibroblasts [23]. p-mTOR was highly expressed in cells within the fibroblastic foci (Fig. 7, upper left and middle left). Unlike Western analysis data, p-mTOR was also expressed in cells in normal alveoli (upper right and middle right). However, LC3-2 expression was barely detectable in the fibroblastic foci derived from IPF patient lung specimens (lower left). In contrast, cells in control lung alveoli specimens expressed easily detectable levels of LC3-2 protein (lower right). Collectively, our results suggest that autophagic activity is also suppressed in myofibroblasts in the fibroblastic foci in IPF patients.

## Discussion

In normal physiological conditions, when tissue injury occurs, fibroblasts become activated, producing extracellular matrix to facilitate the wound healing process and subsequently undergo apoptosis. However, IPF fibroblasts elude this apoptotic process and maintain highly proliferative and apoptotic-resistant properties by using the PTEN/Akt axis, which eventually destroys lung function by producing excessive amounts of extracellular matrix, [15], [16], [34]. A recent study showed that autophagy is not activated in IPF fibroblasts and the inhibition of autophagy promotes extracellular matrix production, suggesting a direct link between the deregulation of autophagy and the fibrotic process [41], [43]. We found that IPF fibroblasts have abnormally low autophagic activity on polymerized collagen. Thus, these observations suggest that IPF fibroblasts have a pathologically altered mechanism to desensitize themselves to collagen matrix driven stress by maintaining low autophagic activity.

**Figure 8:**
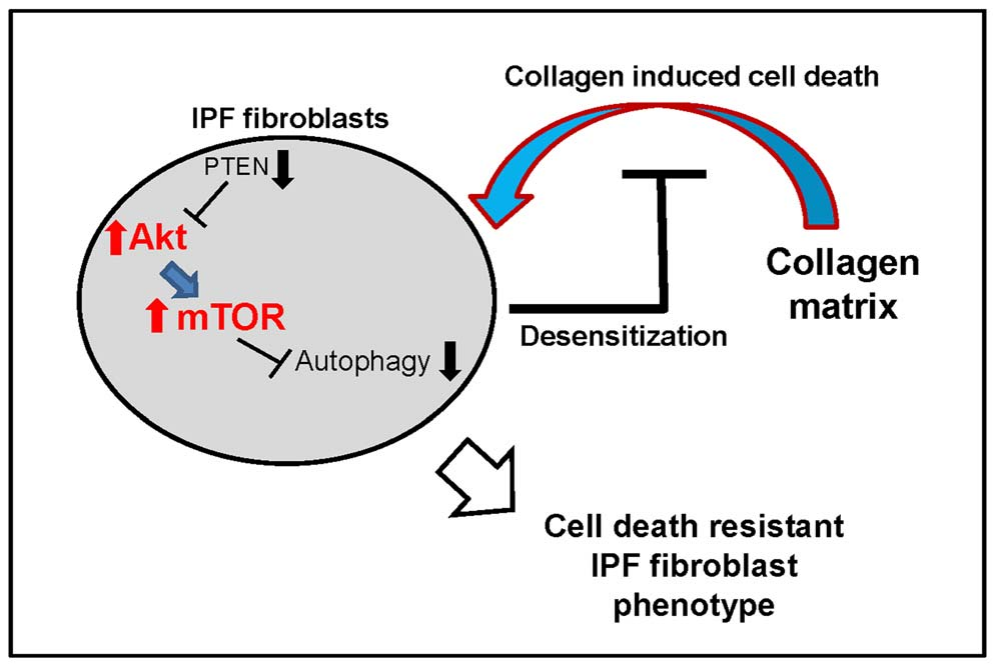
Proposed model for the desensitization to polymerized collagen induced cell death in IPF fibroblasts. We previously found that when control fibroblasts are attached to polymerized collagen, cell death is increased [44]. In contrast, IPF fibroblasts are resistant to collagen matrix induced cell death. In this study, we further elucidate the underlying mechanism that bestows a highly viable IPF fibroblast phenotype in response to polymerized collagen. When IPF fibroblasts are allowed to interact with collagen matrix, Akt activity is inappropriately high due to PTEN suppression, which in turn increases mTOR kinase activity. Autophagy in IPF fibroblasts is then subsequently suppressed, and this pathological event desensitizes IPF fibroblasts to polymerized collagen induced cell death. Upwards arrow indicates enhanced activity. Downwards arrow indicates reduced activity.

We previously found that when IPF fibroblasts attach to collagen rich matrix, Akt activity is up-regulated by PTEN suppression, which subsequently increases cell proliferation [22], [23]. Akt is a crucial kinase regulating mTOR function and LC3-2 expression is inversely regulated by mTOR kinase activity. Thus, our hypothesis was that mTOR kinase activity is aberrantly up-regulated as a result of abnormally high Akt activity in IPF fibroblasts on collagen, inhibiting autophagosome formation. Our experiments demonstrated that IPF fibroblasts are less sensitized to polymerized collagen due to the aberrant PTEN/Akt axis (Fig. 8). In contrast, increased induction of autophagic activity was found as a result of Akt inhibition due to enhanced PTEN activity when control fibroblasts were cultured on collagen matrix.

Cells constantly monitor their surrounding environment to maintain their homeostasis, and autophagy is activated when cells are exposed to an unfavorable/stress causing environment. [31]–[33], [40]. Thus, it is thought that the inhibition of the protective role of autophagy can sensitize cells to stress, which facilitates cells to be highly susceptible for polymerized collagen driven cell death. A prior study already documented that the inhibition of autophagic function in HeLa cells by 3MA treatment sensitizes them to a serum starved condition, thereby increasing cell death [45]. Likewise, we also found that enhanced cell death occurred when autophagic activity was inhibited in control fibroblasts on collagen matrices. However, the viability of IPF fibroblasts was relatively higher compared with control fibroblasts in the presence of autophagy inhibitors. These results strongly suggest that when IPF fibroblasts attach to polymerized collagen matrix, enhanced Akt/mTOR kinase activities suppress autophagy induction, and that further inhibition of already low autophagy has a minimal effect on cell viability. However, this finding can be reversed when PTEN is re-introduced or Akt is inhibited in the presence of autophagy inhibitors in IPF fibroblasts. These data show that the altered PTEN/Akt/mTOR axis suppresses autophagic induction when IPF fibroblasts are cultured on collagen, permitting IPF fibroblasts to maintain viability on collagen. Unlike IPF fibroblasts, we previously found that enhanced cell death occurs when control cells were cultured on collagen for an extensive time period [44]. Thus, it is thought that control fibroblasts sense polymerized collagen as a stress, and that autophagy is initially used as a defense mechanism to protect them from collagen matrix induced cell death until it reaches its threshold. However, IPF cells maintain low autophagy on collagen and this aberrant function permits IPF fibroblasts to maintain their viability.

Our results suggest that there are biological differences in LC3-2 and mTOR kinase activities in IPF and control fibroblasts. To address this, we analyzed multiple control and IPF fibroblasts cultured on polymerized collagen. We found that IPF fibroblasts have abnormally low autophagic activity in response to collagen matrix and this is due to an altered PTEN/Akt/mTOR axis. Our results were also supported from another group's prior finding that autophagic activity is reduced in IPF fibroblasts [41]. In our current study, we further demonstrate that IPF fibroblasts acquire additional pathological properties by suppressing PTEN/Akt/mTOR-dependent autophagic activity, which permits IPF fibroblasts to efficiently maintain their viable phenotype on collagen rich matrix. Although our results indicate that IPF fibroblasts maintain low autophagy on polymerized collagen due to the deregulation of mTOR, we cannot rule out the possibility that an mTOR-independent mechanism to suppress LC3-2 expression exists. Because biological heterogeneity exists in patient specimens due to various ages, health, sex, and life styles (smoking, etc), it is a plausible assumption that each specimen is not biologically identical. Nevertheless, our main goal of this study was to examine whether enhanced Akt/mTOR suppresses LC3-2 expression in most IPF fibroblasts on collagen and we found that when IPF fibroblasts interact with collagen rich matrix, the alteration of a Akt/mTOR axis can contribute to the desensitization of IPF fibroblasts to the stress inducing effects of polymerized collagen.

Several experiments have previously been conducted to elucidate the role of autophagy on cell death from environmental stress, and our studies are further supported by their findings. For example, autophagy serves as a cell survival mechanism and suppression of autophagy via inhibition of lysosomal function contributes to zVAD-induced necrotic cell death [36]. Also, pharmacological inhibition of autophagy results in increased cell death, suggesting a protective role of autophagy [37]. Furthermore, Akt inhibition promotes autophagy and sensitizes PTEN null tumors to chloroquine [38]. Lamoureux et al. also reported that the autophagy inhibitor chloroquine cooperates with the Akt inhibitor AZD5363 to inhibit autophagy, and sensitizes tumor cells to AZD5363-induced cell death in prostate cancer models [46]. These studies reveal that cells are able to utilize autophagy to protect themselves from stressful environments and the alteration of PTEN/Akt sensitizes cells to cell death- inducing stimuli when autophagic function is inhibited. Likewise, although autophagic activity was low in IPF fibroblasts on collagen, we also found that the modification of the PTEN/Akt axis increased autophagy and the subsequent inhibition of autophagic function significantly promotes IPF fibroblast cell death. Based on our results, it is apparent that IPF fibroblasts elude polymerized collagen driven stress by the alteration of the crucial PTEN/Akt/mTOR pathway and low autophagic activity, which facilitates the desensitization process to polymerized collagen induced cell death. Therefore we propose that the understanding of this regulatory pathway may be a critical event in the response of fibroblast attachment to collagen rich matrix and targeting this axis may be a promising approach for promoting IPF fibroblast cell death. In summary, we found that IPF fibroblasts utilize the PTEN/Akt/mTOR-dependent autophagic regulatory pathway to elude the cell death inducing effect of polymerized collagen, and the alteration of the PTEN/Akt axis and the inhibition of autophagic activity may be an effective approach to sensitize IPF fibroblasts to collagen matrix and a useful strategy to limit the progression of IPF.

## Supporting Information

Figure S1 **Inverse expression patterns of p-mTOR and LC3-2 in each control and IPF fibroblast cultured on collagen.** A) Protein expression of p-mTOR and LC3-2 normalized to GAPDH of each individual control fibroblast was plotted. B) Protein expression of p-mTOR and LC3-2 normalized to GAPDH of each individual IPF fibroblast was plotted. Note that most control fibroblasts have low p-mTOR level while LC3-2 expression is elevated. In contrast, the majority of IPF fibroblasts have enhanced p-mTOR expression while LC3-2 level is low. Protein expression of each p-mTOR and LC3-2 was analyzed from the same control or IPF fibroblasts tested in Fig 1A and Fig 2A.
(TIFF)

Figure S2 **Suppression of autophagic activity using 10 µM of 3MA and/or CQ in IPF fibroblasts over-expressing PTEN or dominant negative Akt increases IPF fibroblast cell death on collagen.** A) 3×10^4^ IPF fibroblasts infected with adenovirus expressing wild type PTEN (WP), dominant negative Akt (DA), or empty vector (GFP) were cultured on polymerized collagen in the presence of 10 µM of 3MA or DMSO in serum free medium and viable cells were measured at 24 h. *p*<0.03 versus GFP. B) IPF fibroblasts infected with adenovirus expressing wild type PTEN (WP), dominant negative Akt (DA), or empty vector (GFP) were cultured on polymerized collagen in the presence of 10 µM of CQ or water in serum free medium and viable cells were measured at 24 h. C) IPF fibroblasts infected with adenovirus expressing wild type PTEN (WP), dominant negative Akt (DA), or empty vector (GFP) cultured on polymerized collagen matrix were treated with 10 µM of 3MA and CQ together (3MA+CQ) and viable cells were measured at 24 h. *p*<0.04 versus GFP, *p*<0.02 versus DMSO+Water as indicated. All assays were performed in triplicate using IPF cells shown in lane 5 in an upper panel in Fig 1A.
(TIFF)
