# Supplementary figures and images for: IPF Fibroblasts Are Desensitized to Type I Collagen Matrix-Induced Cell Death by Suppressing Low Autophagy via Aberrant Akt/mTOR Kinases
Source: PLoS One. 2014 Apr 11;9(4):e94616. doi: 10.1371/journal.pone.0094616 (PMC3984186; doi:10.1371/journal.pone.0094616)

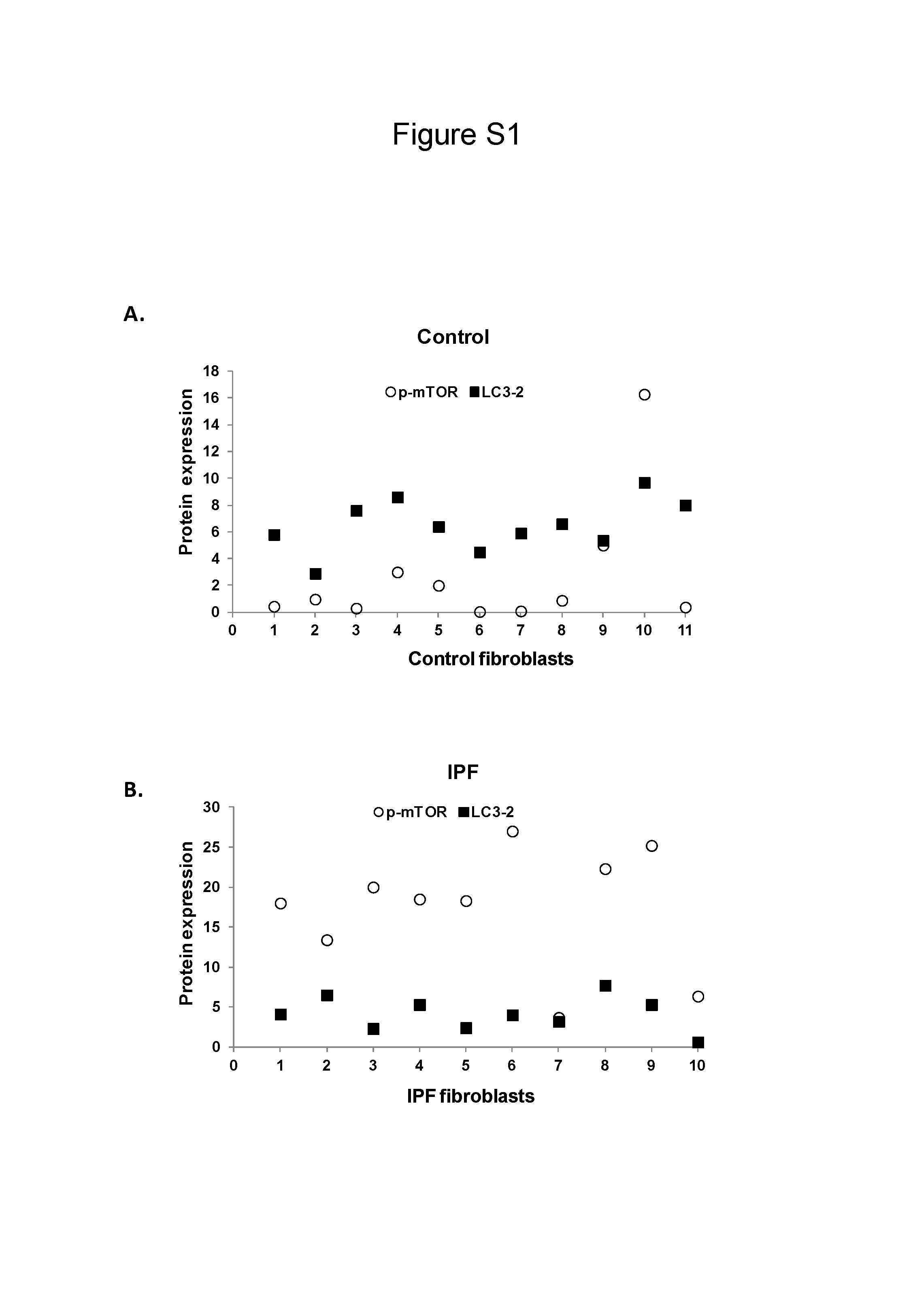

Supplement: Figure S1 — Inverse expression patterns of p-mTOR and LC3-2 in each control and IPF fibroblast cultured on collagen. A) Protein expression of p-mTOR and LC3-2 normalized to GAPDH of each individual control fibroblast was plotted. B) Protein expression of p-mTOR and LC3-2 normalized to GAPDH of each individual IPF fibroblast was plotted. Note that most control fibroblasts have low p-mTOR level while LC3-2 expression is elevated. In contrast, the majority of IPF fibroblasts have enhanced p-mTOR expression while LC3-2 level is low. Protein expression of each p-mTOR and LC3-2 was analyzed from the same control or IPF fibroblasts tested in Fig 1A and Fig 2A. (TIFF) [file pone.0094616.s001.tiff]

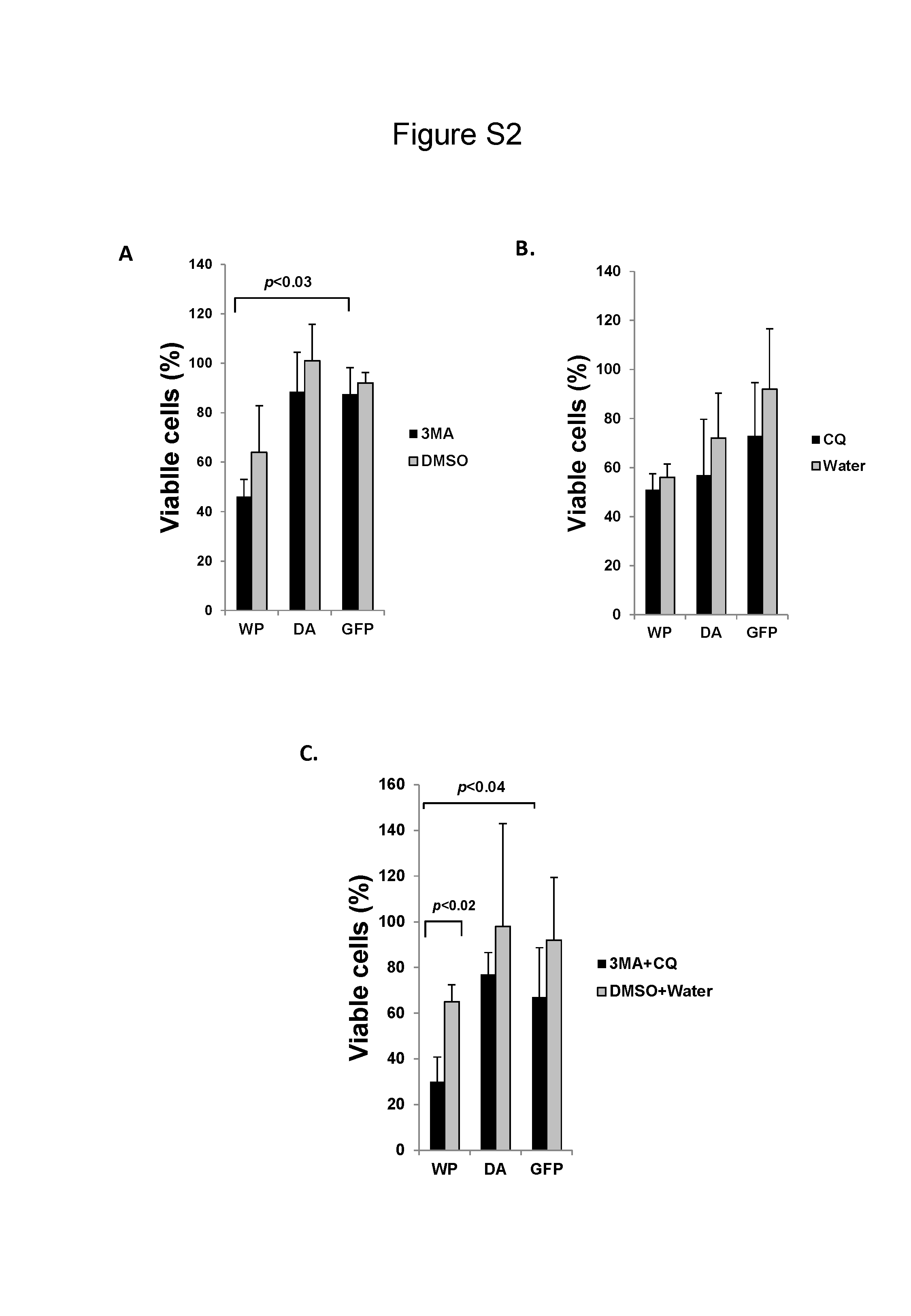

Supplement: Figure S2 — Suppression of autophagic activity using 10 µM of 3MA and/or CQ in IPF fibroblasts over-expressing PTEN or dominant negative Akt increases IPF fibroblast cell death on collagen. A) 3×10^4^ IPF fibroblasts infected with adenovirus expressing wild type PTEN (WP), dominant negative Akt (DA), or empty vector (GFP) were cultured on polymerized collagen in the presence of 10 µM of 3MA or DMSO in serum free medium and viable cells were measured at 24 h. p<0.03 versus GFP. B) IPF fibroblasts infected with adenovirus expressing wild type PTEN (WP), dominant negative Akt (DA), or empty vector (GFP) were cultured on polymerized collagen in the presence of 10 µM of CQ or water in serum free medium and viable cells were measured at 24 h. C) IPF fibroblasts infected with adenovirus expressing wild type PTEN (WP), dominant negative Akt (DA), or empty vector (GFP) cultured on polymerized collagen matrix were treated with 10 µM of 3MA and CQ together (3MA+CQ) and viable cells were measured at 24 h. p<0.04 versus GFP, p<0.02 versus DMSO+Water as indicated. All assays were performed in triplicate using IPF cells shown in lane 5 in an upper panel in Fig 1A. (TIFF) [file pone.0094616.s002.tiff]
